# Influence of shear stress magnitude and direction on atherosclerotic plaque composition

**DOI:** 10.1098/rsos.160588

**Published:** 2016-10-19

**Authors:** Ryan M. Pedrigi, Vikram V. Mehta, Sandra M. Bovens, Zahra Mohri, Christian Bo Poulsen, Willy Gsell, Jordi L. Tremoleda, Leila Towhidi, Ranil de Silva, Enrico Petretto, Rob Krams

**Affiliations:** 1 Department of Bioengineering, Imperial College London, London, UK; 2 National Heart and Lung Institute, Imperial College London, London, UK; 3 MRC-Clinical Sciences Centre, Imperial College London, London, UK; 4 Institute of Clinical Medicine and Department of Cardiology, Aarhus, Denmark; 5 Duke-NUS Medical School, Singapore, Republic of Singapore; 6 Centre for Trauma Sciences, Queen Mary University of London, London, UK; 7 Biomedical MRI, Department of Imaging and Pathology, KU Leuven, Leuven, Belgium

**Keywords:** atherosclerosis, biomechanics, haemodynamics, endothelial cell, micro-computed tomography, thin cap fibroatheroma

## Abstract

The precise flow characteristics that promote different atherosclerotic plaque types remain unclear. We previously developed a blood flow-modifying cuff for ApoE^−/−^ mice that induces the development of advanced plaques with vulnerable and stable features upstream and downstream of the cuff, respectively. Herein, we sought to test the hypothesis that changes in flow magnitude promote formation of the upstream (vulnerable) plaque, whereas altered flow direction is important for development of the downstream (stable) plaque. We instrumented ApoE^−/−^ mice (*n* = 7) with a cuff around the left carotid artery and imaged them with micro-CT (39.6 µm resolution) eight to nine weeks after cuff placement. Computational fluid dynamics was then performed to compute six metrics that describe different aspects of atherogenic flow in terms of wall shear stress magnitude and/or direction. In a subset of four imaged animals, we performed histology to confirm the presence of advanced plaques and measure plaque length in each segment. Relative to the control artery, the region upstream of the cuff exhibited changes in shear stress magnitude only (*p* < 0.05), whereas the region downstream of the cuff exhibited changes in shear stress magnitude and direction (*p* < 0.05). These data suggest that shear stress magnitude contributes to the formation of advanced plaques with a vulnerable phenotype, whereas variations in both magnitude and direction promote the formation of plaques with stable features.

## 1. Introduction

Atherosclerosis is a chronic inflammatory disease characterized by the local development of plaques composed of lipids, remnant and apoptotic cells and immune cells within the innermost lining of arteries [1]. Although it is a multifactorial disease, plaques are typically observed in local regions of the vasculature with high curvature or bifurcations and, thus, cannot be explained solely by systemic risk factors such as high cholesterol.

Ample evidence suggests that the non-uniform distribution of plaques is due to altered forces imposed onto the endothelium resulting from blood flow that is often characterized as disturbed or atherogenic and quantified by metrics of wall shear stress (WSS). Studies in patients have identified that plaque progression occurs within regions experiencing changes in the magnitude and/or direction of WSS [2,3], but there is debate as to which flow disturbance is most responsible for the development of advanced plaques [4–6].

To examine this issue further, we previously developed a tapered cuff for placement around one of the carotid arteries of ApoE^−/−^ mice to induce several regions of flow disturbance within the artery, including low, high and oscillatory flow. These flow disturbances caused the development of atherosclerotic plaques upstream and downstream of the cuff that contained features of advanced plaques such as an abundance of lipids and macrophages. In addition, upstream plaques had a higher concentration of lipids, and matrix metalloproteinases overlaid by a thin and inflamed fibrous cap, similar to vulnerable plaques in humans (as determined by an independent expert human pathologist [7]), whereas downstream plaques resembled a more stable plaque phenotype [7]. This model has been shown to reproducibly promote the formation of advanced plaques with vulnerable and stable features in many additional studies by us [8–12] and others [13–16].

Despite the wide use and importance of this animal model for studying experimental advanced plaques, no study has systematically characterized the flow profiles that result from cuff placement *in vivo*, which may aid in understanding the discrepant results between studies as to which flow profile promotes each advanced plaque type [4,5]. We hypothesize that vulnerable plaques are promoted by low WSS magnitude, whereas stable plaques are promoted by altered WSS direction. Herein, we evaluated this hypothesis using our mouse model of advanced plaques by showing, for the first time, to the best of our knowledge, evaluation of six WSS metrics of disturbed flow, two of which were recently developed, eight to nine weeks after cuff placement when advanced plaques are fully developed.

## 2. Methods

### 2.1. Animals and surgery

Animal care and all experimental procedures complied with the Animals (Scientific Procedures) Act of 1986. Female ApoE^−/−^ mice (20–23 g in weight) were placed on a high-fat Western-type diet (Lillico Biotech, UK) at the age of 11 weeks. Two weeks later, each mouse was anaesthetized with an intraperitoneal injection of Domitor (medetomidine, 1 mg ml^−1^, Orion Pharma), hypnovel (Midazolam, 5 mg ml^−1^, Roche), vetergesic (buprenorphine, 0.1 mg ml^−1^, Alstoe Animal Health) and saline in ratios proportional to their body weight and then instrumented with a blood flow-modifying cuff (Promolding, The Netherlands) around the left carotid artery that tapers from 500 µm at the inlet to 250 µm at the outlet.

### 2.2. *In vivo* imaging and three-dimensional vessel reconstruction

Mice were scanned with micro-CT between eight and nine weeks after cuff placement (*n* = 7 cuffed arteries and *n* = 7 control arteries) to obtain an accurate three-dimensional reconstruction of the *in vivo* geometry of each carotid artery. Prior to each scan, animals were anaesthetized, using isoflurane (5% for induction and 1.5% for maintenance in 100% oxygen at 1.5 l min^−1^), hair was removed from the chest and base of the hind limb, and ECG paediatric surface electrodes (3M, USA) were taped to the skin. A bolus injection of 100 µl Viscover ExiTron nano 12 000 (Miltenyi Biotec, Germany), an alkaline earth metal-based nanoparticle contrast agent, was then intravenously administered via the tail vein. This blood-pool contrast agent provides high contrast for 4–8 h, significantly improving visualization of the vasculature in mice with a small injection volume owing to its high metal load. The mouse was then placed on a custom bed equipped with a face mask, respiratory pillow (M2M, USA) and electrical heating pad (37°C) and centred in the micro-CT field of view using laser alignment (Siemens Inveon, Germany). The field of view extended from the aortic arch to the carotid artery bifurcations and included both the internal and external carotid arteries.

To reduce radiation exposure and facilitate follow-up scanning, the scanner mode was converted from conventional helical scanning to step-and-shoot mode with a settle time of 150 ms (this conversion also limited the vibration induced by the rotation of the gantry, hence minimizing motion blurring artefacts). Helical scanning acquires images over the entire cardiac cycle and gating is done retrospectively, whereas the step-and-shoot mode acquires images only at specific points in the cardiac cycle (triggered by the R-peak in the ECG trace) and thus greatly reduces the radiation exposure by reducing the overall number of acquired images [17]. Each mouse was scanned for approximately 35 min. The micro-CT scan projections were reconstructed, using a filtered back-projection-based method modified from the Feldkamp algorithm with no downsampling, a bilinear interpolation and a Shepp-Logan filter using proprietary software (COBRA Exxim, Exxim Computing Corp., USA). Polynomial-based soft tissue beam hardening correction was applied to the projection data for micro-CT angiography, and images were exported as DICOM files.

DICOM files were loaded into the openware image analysis software package vascular modelling toolkit (VMTK) [18], and segmentation was performed as follows. The colliding front's method of initialization was used to propagate the boundary of the vessel between two manually placed seed points, one at each end of the selected arterial segment, which included all pixels within the threshold limits specified. In some cases, signal intensities varied along the length of the artery requiring the method to be applied over sections of the vessel with different thresholds and merging the different segmented sections together to obtain the complete initialized model of the artery. The output from the model initialization was an image that was extracted, using a marching cube algorithm. Because the extracted lumen surface was often uneven, a Taubin smoothing step was used to even out the bumps on the surface that were not physiologically realistic while taking care not to artificially shrink the lumen surface by over-smoothing. In the majority of cases, the surface was smoothed with 100 iterations and a spatial band pass of 0.03 internal VMTK units. Finally, the ends of the three-dimensional model were clipped orthogonal to the vessel centreline and exported as an STL file for computational fluid dynamics (CFD) simulations.

### 2.3. Phantom validation of the imaging workflow

**Figure 1.**
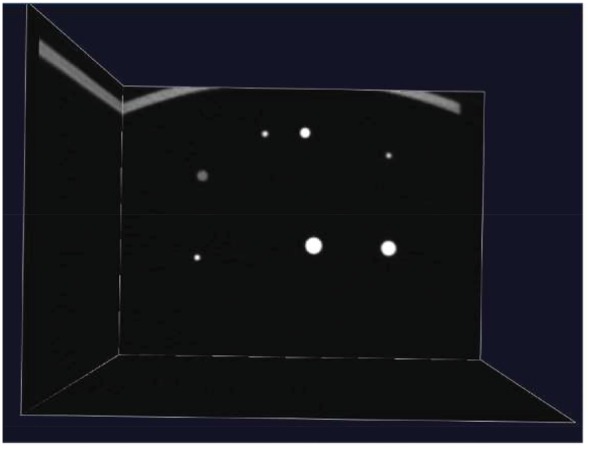
Micro-CT image shows the cross sections of seven glass capillaries filled with contrast agent of varying dilutions (which caused altered image intensity) and varying diameters, including 0.5 mm (*n* = 3), 1.0 mm (*n* = 2) and 1.5 mm (*n* = 2). In this study, we evaluated only the error associated with the 0.5 mm diameter capillaries as this is most representative of the size of the mouse carotid artery (which is approximately equivalent in diameter).

We validated the accuracy of the *in vivo* imaging workflow, segmentation and reconstruction of the vessel geometry by imaging three glass capillaries with an internal diameter of 500 µm (figure 1). These phantoms were comparable in size to the mouse carotid arteries, which had mean inlet diameters of 622 ± 17 and 594 ± 13 µm for the left and right arteries, respectively. The capillaries were filled with contrast agent and imaged using the same approach (micro-CT scanner, image processing software and settings) as that used to image the mouse carotid arteries. The mean percentage error in diameter over the three capillaries was 4.5 ± 2.4% or approximately 28 µm, which is less than one pixel (micro-CT resolution was 39.6 µm).

### 2.4. Doppler blood velocity measurements

Ultrasound measurements of blood velocity at the inlet of each carotid artery within each of the imaged mice were conducted 1–3 days after micro-CT imaging. Blood velocity was also recorded in two separate mice at baseline (i.e. before cuff placement). Each animal was anaesthetized using isoflurane (2% inhalation) and placed on a custom bed in a supine position with its paws taped to the ECG electrodes for direct contact. The body temperature of the animal was continuously monitored using a rectal probe and adjusted when necessary (Indus Instruments, USA). Hair from the neck was removed, and the region was covered with a layer of pre-warmed ultrasound gel (Aquasonic 100, Parker, USA). Ultrasound was performed using the high-resolution imaging system Vevo 770 (Visualsonics, Canada) with an RMV707B transducer and a broadband frequency of up to 45 MHz.

Prior to taking the velocity measurements, B-mode imaging was used to identify an anatomical landmark (the right subclavian branch in the right artery and the aortic arch in the left artery) proximal to which the measurements were made. Pulsed Doppler was used to measure the blood velocity in both the left and right carotid arteries immediately downstream of this anatomical point within each mouse. The Doppler imaging sample volume size was adjusted according to the vessel diameter, and it was placed in the centre of the vessels. To obtain accurate blood velocity measurements, the vessel being imaged was aligned as parallel as possible to the direction of the ultrasound beam (which minimized the angle of incidence between the ultrasound beam and the blood flow direction in the imaged vessel). For all cases, the beam incidence angle was maintained below 60°. All data were converted to axial (e.g. vessel direction) velocities using the cosine of the angle.

### 2.5. Computational fluid dynamics

The mesh of each three-dimensional reconstructed carotid artery geometry was composed of the common carotid artery (inlet), the carotid bifurcation and the initial region of the internal (outlet 1) and external (outlet 2) carotid arteries. Flow extensions were added to the inlet and outlet at a length of 1.5 and 7 times their diameters, respectively, which has been demonstrated to allow for fully developed flow [19]. Meshing was performed in Gambit v. 2.4.6 (Fluent, Ansys, USA). The surface of the flow extensions was meshed with quadrilateral elements, whereas the surface of the common carotid artery and branches was meshed with triangular elements with a maximum edge length of 0.04 mm. A boundary layer consisting of six rows of prismatic elements with an initial height of 0.005 mm and growth rate of 1.2 was added to the walls of the geometry to increase the mesh density close to the wall and accurately solve for WSS. The volume of the common carotid and internal and external branches were meshed with tetrahedral elements using the automatic meshing scheme in Gambit. A total of 14 arterial geometries were meshed and a convergence test demonstrated mesh independence of each geometry on the solution.

Each meshed artery was then imported into the CFD package Fluent v. 6.3 (Ansys) to numerically solve the Navier–Stokes equations of motion for a fluid. Properties of the simulated fluid and boundary conditions were also prescribed within the model. Blood was assumed to be an incompressible Newtonian fluid with a viscosity of 3.5 mPa s [20] and density of 1050 kg m^−3^ [21]. The Newtonian fluid assumption is justified as mean shear rates are greater than 1000 s^−1^ in both the instrumented and control mouse carotid arteries (the Reynolds number is approx. 40), where Newtonian and non-Newtonian models of blood behave similarly [22]. Simulations imposed a pulsatile flow of the blood at the inlet of the vessel by assigning a plug flow profile equal to the peak velocity measured at each point in the cardiac cycle, as has been reported previously [23]. The corresponding velocity wave form represented the average wave form over all cardiac cycles acquired with Doppler ultrasound. The extended inlet allowed the flow to become fully developed before entering into the actual lumen reconstruction. The outlet of the vessel had a prescribed pressure of 0 mmHg. The lengthy outflow extension (approx. 4 mm) allowed the flow to reach a stable state before exiting the computational domain, which limited backflow (backflow was only observed in the first cycle of the simulations, but not during the second or third cycles). The lumen surface was assumed to be stationary with a no-slip boundary condition.

Surface monitors were created to monitor the velocity magnitude at the inlet (U) and WSS throughout the simulation. Convergence was achieved when the residuals fell below 10^−5^ (m s^−1^) at each time step in the transient simulation. Every simulation was run over three cardiac cycles, where data from the first two cycles were excluded from the analysis to ensure independence from the initial conditions. Shear stress obtained from the second cycle was found to be within 1% of that in the third cycle, demonstrating convergence. During the third cardiac cycle, blood velocity and shear stress data were exported at every 10th time step (out of 300) for post-processing, meaning these metrics were evaluated at a total of 30 points over the cardiac cycle (i.e. because the mouse heart rate was 8 Hz or 0.125 s per cardiac cycle and the flow velocity inputted to the CFD simulation was divided into 300 points or time steps (0.00042 s per time step), blood velocity and shear stress were evaluated at every 10th time step or every 0.0042 s over the cardiac cycle). The Courant–Friedrichs–Lewy number used in these simulations was a maximum of 0.45.

### 2.6. Quantifying shear stress metrics of atherogenic flow

The CFD simulation resulted in a WSS vector (magnitude and direction) that was imported into a custom Matlab program (R2012a, MathWorks, USA) to quantify metrics of atherogenic flow. These metrics were chosen because they have been demonstrated to localize to regions of atherosclerotic plaque development while each quantifying a different aspect of the flow field as a function of WSS magnitude, direction or both. These metrics include the wall shear stress angle deviation (SAD; a metric of the spatial change in flow direction) [24], relative residence time (RRT; a metric of species transport) [25], oscillatory shear index (OSI; a metric of flow reversal) [26] and the recently developed transverse wall shear stress (tSS; a metric of flow multidirectionality) [27,28]. In addition to these established metrics, we developed two new WSS metrics, the low shear index (LSI) and the high shear index (HSI) [1], which quantify changes in time-averaged WSS (TAWSS) magnitude on a continuous scale. They are formulated as

$$\mathrm{LSI}=\frac{{\mathrm{TAWSS}}_{\mathrm{LowThresh}}-\mathrm{TAWS}S_{\mathrm{Instrumented}}}{\mathrm{TAWS}S_{\mathrm{LowThresh}}},$$

where

2.1 $$\mathrm{TAWS}S_{\mathrm{LowThresh}}=e^{\left[\bar{\ln(\mathrm{TAWS}S_{\mathrm{Control}})}-0.67\cdot \mathrm{SD}(\ln(\mathrm{TAWS}S_{\mathrm{Control}}))\right]}$$

and

$$\mathrm{HSI}=\frac{\mathrm{TAWS}S_{\mathrm{Instrumented}}-\mathrm{TAWS}S_{\mathrm{HighThresh}}\;}{\mathrm{TAWS}S_{\mathrm{Instrumented}}},$$

where

2.2 $$\mathrm{TAWS}S_{\mathrm{HighThresh}}=e^{\left[\bar{\ln\mathrm{TAWS}S_{\mathrm{Control}}}+0.67\cdot \mathrm{SD}(\ln(\mathrm{TAWS}S_{\mathrm{Control}}))\right]}$$

The thresholds for non-zero values of LSI (TAWSS_LowThresh_) and HSI (TAWSS_HighThresh_) were determined by normalizing, via log-transformation, the mean TAWSS values over the contralateral control vessel (the mean is indicated by the overbar in the formulation), which is assumed to represent non-atherogenic blood flow, and computing the bottom and top 25% of the distribution (equal to 0.67 × standard deviation) for each metric, respectively. Exponential transformation of these two values was used to obtain the values of TAWSS for discriminating atherogenic low and high TAWSS. Values of TAWSS at each point in the instrumented artery were then compared with these threshold values to characterize the degree of low or high TAWSS on a continuous scale (to obtain a value for LSI and HSI in the control artery, this same threshold was compared with each point in the control vessel). This definition of low and high WSS allows for a window of normal WSS, thus the absence of one metric does not imply presence of the other.

To facilitate comparison between the instrumented and contralateral control vessels, the mean of each metric (over the seven mice imaged) in both of these vessels is divided by the mean of the given metric in the control vessel (thus, the mean value of each metric in the control is always 1 and that in the instrumented vessel is a factor of the value in the control). To avoid dividing by zero (or near zero values, causing ballooning of the ratio), metrics near or at zero were set to the very small but non-zero value of 0.005. The instrumented vessel is segregated into four regions: upstream of the cuff outside of the vulnerable plaque region (determined by the absence of plaques), upstream of the cuff within the region of vulnerable plaque (from the cuff entrance to 1.6 mm upstream of the cuff entrance, based on plaque length measurements from histology and accounting for vessel shrinkage), within the cuff and downstream of the cuff within the stable plaque region (the control vessel is similarly divided into regions based on an equivalent distance from the end of the vessel as the regions determined in the instrumented vessel). To facilitate visual comparisons in the distributions between WSS metrics, metrics were scaled from 0 (null value) to 1 (maximum value, which represented the maximum flow disturbance of the type represented by that metric) by dividing all values by the theoretical maximum for the given metric (except RRT, which was divided by its maximum over all mouse vessels).

### 2.7. Histology

Immediately after Doppler ultrasound measurements were taken (1–3 days after micro-CT imaging), four of the mice were euthanized for histological evaluation of plaque length. An additional nine mice were also euthanized nine weeks after cuff placement for histological analysis, but they were not imaged. In all mice evaluated for histology (*n* = 13 total), euthanasia was performed by intraperitoneal injection with an anaesthetic mix, followed by perfusion fixation (10 ml PBS followed by 10% formalin). The cuff was carefully removed from the left carotid artery, and both carotid arteries were collected, including the aortic arch and carotid bifurcation. Arteries were placed in OCT medium and frozen at −20°C. Frozen carotid tissue was sectioned at 8 µm thickness, and sections were collected over the entire vessel length, including the aortic and carotid bifurcations, which allowed evaluation of plaque length.

A lipid stain was performed to better visualize the plaque. Lipids were detected with Oil-red-O staining (0.5% Oil-red-O solution in Propylene glycol; Sigma Aldrich, UK) and Mayer's haemotoxylin (Sigma Aldrich) was used as a nuclear counterstain. After staining, all histology sections were imaged (MicroBeam 4.5 Pro laser capture microscope; Carl Zeiss, UK) at 10× magnification (the microscope and settings were kept the same for all stains across all mice). The lipid stain was used only for visualization of plaque length, not for characterization of plaque type which has been done previously in this animal model [7,12].

### 2.8. Statistical analysis

Data are reported as mean ± s.e. Blood velocity and vessel diameter comparisons were performed with a one-way ANOVA. Comparisons for each metric of atherogenic flow between instrumented and control vessels were performed with a one-way student's *t*-test, because we were only interested in whether each flow metric was larger in the instrumented vessel compared with control, and no comparisons between metrics were performed. The threshold of statistical significance was set at *p* < 0.05.

## 3. Results

**Figure 2.**
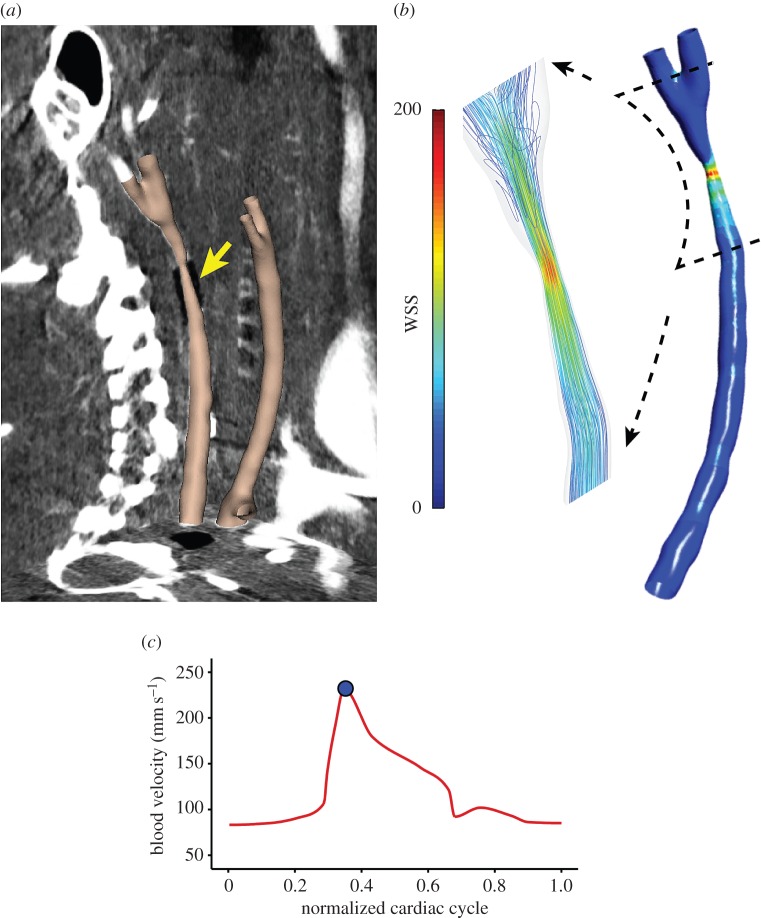
(*a*) Final three-dimensional reconstruction of both carotid arteries, instrumented and control, from a representative mouse, that were digitally flesh-coloured and superimposed onto the three orthogonal planes of the raw micro-CT image dataset. The position of the cuff is indicated by the yellow arrow. (*b*) Distribution of WSS magnitude (Pa) within the instrumented vessel from CFD. The magnified inset shows streamlines around the region of the cuff (note the presence of multidirectional flow in the downstream segment). (*c*) Line plot shows the pulsatile blood velocity (mm s^−1^) measured over the cardiac cycle of the instrumented vessel, which was imposed at the inlet of the model in the CFD simulation used to compute WSS (blue dot indicates the position in the cardiac cycle as peak systole for the given WSS map).

The carotid arteries of the imaged mice could be clearly demarcated within the micro-CT images owing to the contrast agent, and the radiopaque cuff could be seen attached to the instrumented vessel (figure 2*a*). The constrictive cuff created a flow-limiting areal stenosis of approximately 75%, which reduced blood flow in the upstream segment, increased flow within the cuff, and created a mixture of low, high and multidirectional flow downstream of the cuff owing to a time-varying vortex (figure 2*b*). Blood velocity measured by ultrasound at the inlet of the un-instrumented mice at baseline had a mean of 172.8 ± 3.6 mm s^−1^ (*n* = 4 vessels from two mice). Placement of the cuff caused a significant lowering of blood velocity to a mean of 112.8 ± 13.7 mm s^−1^ (*n* = 7), which was statistically lower than baseline (*p* < 0.05) and lower than the velocity at the inlet of the contralateral control vessels within those mice (which had a mean velocity of 153.4 ± 11.9 mm s^−1^; *n* = 7; *p* < 0.05). Velocities at the inlet of the control versus baseline vessels were not statistically different (*p* = 0.27). In addition, there was no statistical difference between the mean inlet diameter of the control vessels (596.3 ± 16.0 µm) compared with the instrumented vessels (643.1 ± 24.2 µm; *p* = 0.13).

**Figure 3.**
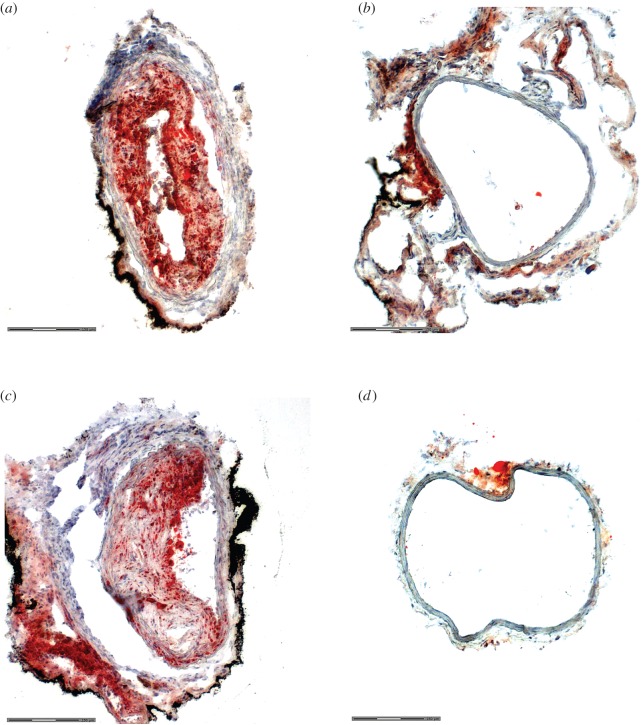
Representative histological sections from the instrumented and contralateral control arteries of a representative mouse at nine weeks, stained for lipids. Three sections are shown from the instrumented artery: (*a*) upstream of the cuff in the region of plaque (with vulnerable phenotype), (*b*) within the cuff (no plaque) and (*c*) downstream of the cuff (stable plaque region). (*d*) The control artery exhibited no plaque development. Sections are shown to demonstrate plaque development, but the characterization of plaque type was performed in previous studies [7,12].

The flow disturbances induced by the cuff caused plaque development upstream and downstream of the cuff (figure 3). No lesions developed in either the cuff region of the instrumented vessel or the contralateral control. In the upstream segment, plaques were 1.5 ± 0.2 mm in length (*n* = 13; values are given as the *in vivo* equivalent length via adjusting the measurements obtained from histology by the mean vessel shrinkage of 43.5 ± 1.5%). Plaques were present over nearly the entire downstream segment with an average length of 1.2 ± 0.1 mm. Previous work established that plaques upstream of the cuff have vulnerable features, whereas those downstream more closely resemble stable lesions [7–16].

**Figure 4.**
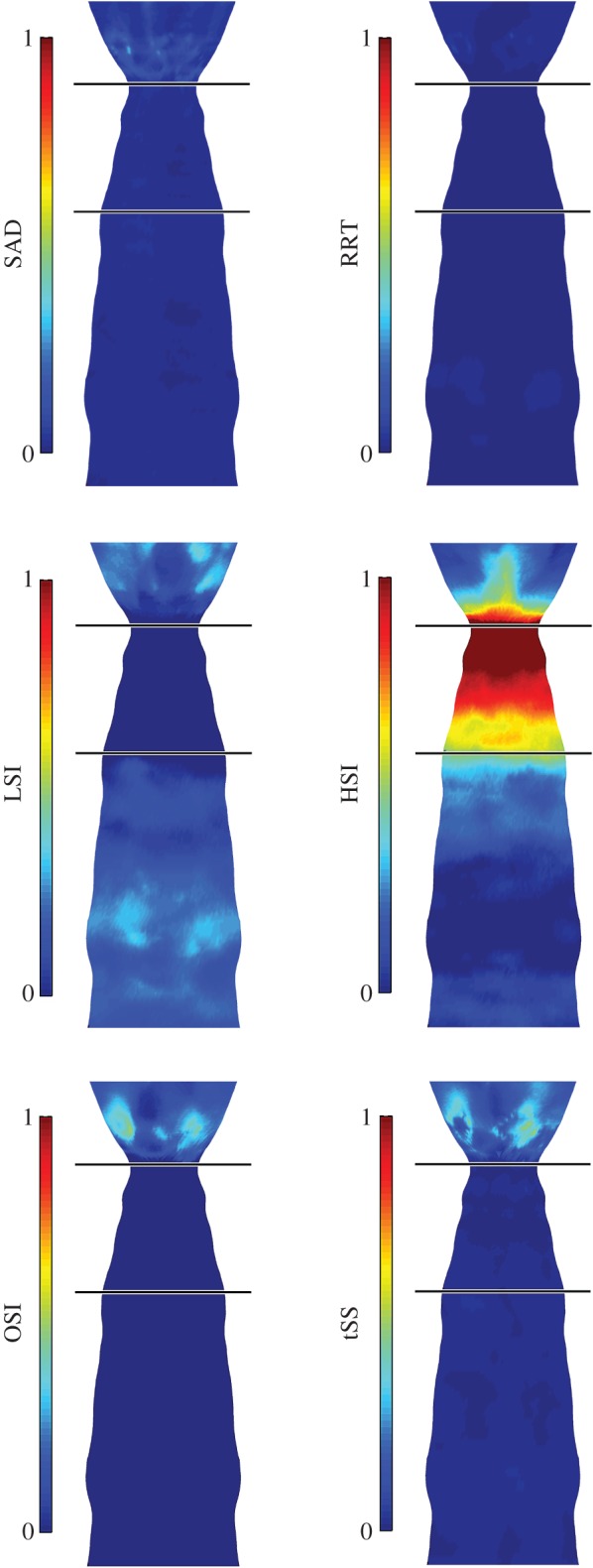
‘En face’ displays show the distribution of each WSS metric of atherogenic flow (averaged over the seven imaged mice and mapped onto one representative instrumented carotid artery geometry). In these displays only, each WSS metric is normalized by its maximum theoretical value to facilitate comparisons between metrics, thus all metrics range from 0 (no value) to 1 (maximum value). The cuff is designated by the black horizontal lines, and the carotid bifurcation is not displayed. The direction of blood flow is from bottom (upstream, proximal to the aortic bifurcation) to top (downstream, towards the carotid bifurcation).

**Figure 5.**
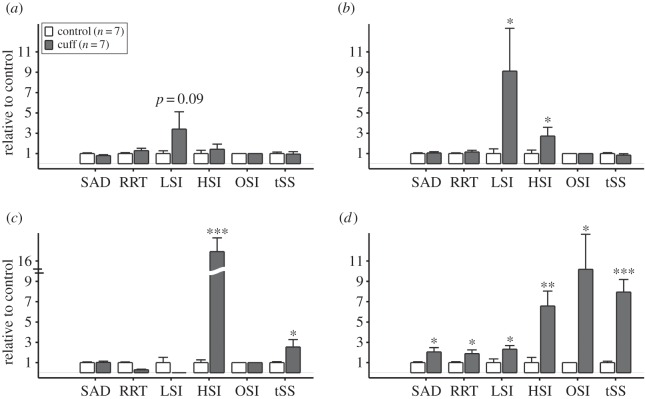
The mean magnitude of each WSS metric averaged over all instrumented (*n* = 7; dark grey bars) and contralateral control (*n* = 7; white bars) vessels segregated into (*a*) upstream outside of the plaque, (*b*) upstream within the plaque, (*c*) cuff and (*d*) downstream regions (each control vessel was segregated into the same number of vessel segments of comparable length as the contralateral instrumented vessel for purposes of comparison). The values of each WSS metric for both the instrumented and control vessels are scaled by the mean of the WSS metric over the control vessels (thus, all controls have a mean scaled value of 1). Comparisons were made only between instrumented and control for each WSS metric. Statistically significant differences are indicated by *p < 0.05, **p < 0.01, and ***p < 0.001. Bars are mean ± s.e.

The vessel geometry from micro-CT (figure 2*a*) and blood velocity from Doppler-ultrasound (figure 2*c*) allowed calculation of the vectored WSS distributions within each mouse carotid artery using CFD (figure 2*b*). The results from CFD served as the basis for computing six WSS metrics of atherogenic flow (figure 4). These metrics were evaluated to characterize different aspects of the flow disturbance within each of the four regions of the instrumented vessel: upstream of the cuff outside of the plaque, upstream of the cuff within the plaque, within the cuff, and downstream of the cuff (the entire downstream region contained plaque). In the vessel segment upstream of the cuff, the WSS metrics that characterize changes in flow direction had negligible values owing to the low Reynolds number (40–80) and the lack of bifurcations or other mechanisms that could induce such flow disturbances. Here, the metrics that exhibited the largest relative values were those that quantified variations in WSS magnitude, LSI and HSI. In the upstream segment outside of the region of plaque development, there was an increase in low WSS (LSI) compared with the contralateral control (*n* = 7; *p* = 0.09; figure 5*a*), whereas within the region of plaque development both low (LSI) and high (HSI) WSS were significantly increased (*p* < 0.05; figure 5*b*). As expected, high WSS (HSI) was dominant within the tapered cuff region with values substantially higher than the control (*p* < 0.001; figure 5*c*). The vessel segment downstream of the cuff experienced changes in both flow magnitude and direction causing all metrics to increase significantly compared with the control (*p* < 0.05), although metrics of oscillatory (OSI) and multidirectional (tSS) flow exhibited the largest increase (*p* < 0.05 and *p* < 0.001, respectively; figure 5*d*).

## 4. Discussion

We have previously characterized the WSS profiles in this ApoE^−\−^ mouse model of disturbed flow-induced atherosclerosis *ex vivo*, demonstrating low WSS in the upstream segment and low and oscillatory WSS in the downstream segment [29]. However, these measurements were made *ex vivo* within one week of cuff placement (before plaque development) and did not evaluate specific WSS metrics of atherogenic flow. Although two other studies have used micro-CT to examine the haemodynamics of the mouse carotid artery [30,31], neither study evaluated flow within the cuff model used herein. We recently reported preliminary data on the distribution of OSI in this model at six and nine weeks based on micro-CT imaging *in vivo*, but did not evaluate other shear metrics [10]. Thus, to the best of our knowledge, this study is the first to evaluate multiple WSS metrics of atherogenic flow within different segments of the mouse carotid artery instrumented with a flow-modifying cuff *in vivo*.

The cuff was designed to induce three regions of flow disturbance: low flow in the segment upstream of the cuff, high flow within the cuff and oscillatory flow in the segment downstream of the cuff. However, changes in lumen geometry after placement of the cuff and development of the resultant plaque may interact with the flow in complex ways to influence final plaque phenotype. Therefore, different metrics were used herein that characterize the flow disturbance using different formulations to account for these potential complexities in the flow field introduced by the cuff.

Each metric characterizes the following aspect of disturbed flow. SAD quantifies the mean angle change in the WSS vector at a point on the lumen wall compared with the neighbouring points, thus examining local *spatial* changes in vector direction; SAD has recently been shown to co-localize with regions of obstructive coronary plaques in patients [32]. OSI quantifies the degree of (180°) *flow reversal* experienced at a point on the vessel wall over the cardiac cycle compared with the direction of the TAWSS vector; it is an established metric that has shown good spatial correspondence with regions of plaque development, in general [26,31,33,34]. One drawback to OSI is that it does not account for WSS magnitude, only back-and-forth directionality. To address this limitation, RRT was developed as a function of both OSI and TAWSS; studies have shown this metric to be a useful correlate to regions of atherosclerosis [31,34,35]. Another drawback to the OSI is that it does not consider deviations in flow direction other than 180° reversal. The tSS metric was developed to account for that limitation. It quantifies the degree of change in flow direction (any direction, except reversal) from that of the TAWSS vector [27]. Because it does not account for flow reversal, it complements the OSI. This metric has recently been shown to correlate well with local regions of naturally occurring early plaques in the aorta of a rabbit model [36] and initiation of advanced plaques in pigs [1]. LSI and HSI quantify the degree of atherogenic low and high WSS, respectively, using a threshold determined by a non-diseased vessel (herein, we used the contralateral control artery within each mouse). Importantly, these metrics quantify low and high WSS over a continuous scale compared with a binary approach used by previous studies [6,19]. These metrics have recently been introduced by us in a previous study where they showed good spatial overlap with advanced plaques, including thin cap fibroatheroma (TCFA), in hypercholesterolemic pigs [1]. Together with the results of this study, these metrics also demonstrate the advantage of scale invariance, where they can be used in animals of different sizes including humans.

Our findings demonstrate that low WSS (quantified by LSI) is prevalent throughout most of the instrumented vessel, which is likely to be an important driver of atherosclerotic plaque development both upstream and downstream of the cuff. Our previous studies demonstrated that plaques in these vessel segments exhibited advanced features and resembled vulnerable and stable plaque phenotypes, respectively [7–16]. In the vessel segment upstream of the cuff outside of the plaque region, LSI was the dominant metric. We were surprised to find that both HSI and LSI were significantly higher within the vulnerable phenotype region of the upstream segment, which is likely to have resulted from positive remodelling of the plaque into the lumen. This finding may be a coincidental phenomenon or it may further promote unstable features within the plaque. Of the remaining WSS metrics, all of which quantify changes in the directionality of the WSS vector, none were present to any significant degree in the upstream segment. This finding aligns with our hypothesis that changes in WSS magnitude (particularly low WSS) are important for the development of vulnerable plaques.

A more complex flow profile existed downstream of the cuff. Here, it was interesting to observe that low WSS (LSI) increased to a much lesser extent and high WSS (HSI) to a much greater extent compared with the upstream vessel segment. More importantly, all of the remaining WSS metrics that quantify changes in flow direction were significantly increased compared with the control vessel, which did not occur in the upstream segment. The largest increase in these metrics was observed with those that quantified flow reversal (OSI) and multidirectionality (tSS) over the cardiac cycle, which increased from the control by a mean factor of 10- and eightfold, respectively. Variations in WSS direction with lumen position (quantified by SAD) were also significantly present, though to a lesser degree (twofold larger compared with the control). Thus, changes in WSS direction over the cardiac cycle (primary) and with spatial location in the lumen (secondary) appear to be the dominant flow disturbances that cause stable advanced plaque development, though a higher magnitude of WSS may also be an important factor.

Our findings align with studies in both mice [7] and pigs [1] which have shown that low WSS is the most prevalent flow disturbance within regions of advanced plaques, including TCFA. In humans, the progression of advanced plaques, in general, has been shown to correlate to regions of low WSS [5,6]; however, it is unclear whether low [4] or high [5] WSS is most important in the final stages of vulnerable plaque development. Our results herein suggest that the discussed discrepancy between low and high WSS in vulnerable plaque development may reflect a difference in timing, wherein low WSS is important for vulnerable plaque initiation and early progression, but in the case of lesions with extensive inward remodelling, high WSS may exacerbate the vulnerable plaque phenotype. In addition to changes in magnitude, the multidirectionality of WSS is also important in the development of atherosclerotic plaques in general, particularly near side branches [26], but few studies have shown a link with advanced plaques at these sites. We recently showed that multidirectional WSS might be important in the initiation of advanced lesions [1]. Our results herein additionally suggest that stable advanced lesions may be promoted by chronic exposure to multidirectional WSS.

### 4.1. Limitations

Three limitations should be considered. First, we employed CFD to model blood flow within the vessels analysed. Although this is a standard approach in the field, it is limited by the assumption of a rigid vessel wall. Future work will seek to employ fluid–structure interaction modelling to consider the elasticity of the artery. Second, we imposed a zero-pressure outlet boundary condition on all CFD simulations. While a mouse-specific outlet boundary condition for each vessel would be ideal, it is not currently feasible to measure the pressure at the vessel outlet *in vivo* given the small size of the vessels (500 µm diameter). Thus, attempting to impose different outlet boundary conditions for each vessel would require guessing the outlet pressure or resistance of the distal vascular beds. Importantly, both carotid arteries link to the circle of Willis (providing redundancy in the cerebral vasculature), which means that the resistance at the outlet of each vessel is expected to be similar. In addition, previous work has found that a zero-pressure outlet boundary condition can accurately reproduce the flow field within a coronary artery [23]. Third, we have chosen to characterize vulnerable plaques in our animal model herein as either plaques with features of vulnerability or plaques with a vulnerable phenotype, to discriminate them from human vulnerable plaques or human TCFAs. Murine plaques in the present model show thin caps, large necrotic cores, minimal VSMC accumulation near the cap and abundantly active immune cells [7,12], but lack the characteristic layering of human TCFAs.

### 4.2. Conclusion

We evaluated the WSS distributions within seven ApoE^−/−^ mice instrumented with a blood flow-modifying cuff *in vivo*. Our findings suggest that changes in WSS magnitude (particularly low WSS) contribute to the formation of advanced plaques with a vulnerable phenotype, whereas changes in WSS magnitude and direction promote the formation of advanced plaques with stable features. Many of the WSS metrics quantifying these flow disturbances within the instrumented vessel were significantly increased, which motivates further work to assess the role of each flow disturbance in promoting each advanced plaque type. Further work is also needed to evaluate the potential of these WSS metrics as prognostic indicators of plaque development. Such work will require the evaluation of precise overlaps between the distribution of each WSS metric, histological markers of advanced plaques (e.g. lipids, macrophages and collagen), and molecular markers of proatherogenic endothelial cell signalling pathways over the course of plaque development.

## Supplementary Material

Figure 5 raw data. The raw values for each shear metric within the instrumented and control arteries of each mouse.

## Data accessibility

The raw values for each shear metric within the instrumented and control arteries of each mouse (figure 5) are available in the electronic supplementary material. All other relevant data are contained within the manuscript.
